# Up-regulation of Myocardial Klotho Expression to Promote Cardiac Functional Recovery in Old Mice following Endotoxemia

**DOI:** 10.21203/rs.3.rs-2949854/v1

**Published:** 2023-05-22

**Authors:** Xueting Li, Yufeng Zhai, Qingzhou Yao, Erlinda The, Lihua Ao, David A. Fullerton, Kai-Jiang Yu, Xianzhong Meng

**Affiliations:** University of Colorado Denve; University of Colorado Denver; University of Colorado Denver; University of Colorado Denver; University of Colorado Denver; University of Colorado Denver; Harbin Medical University Cancer Hospital; University of Colorado Denver

**Keywords:** Klotho, IL-37, inflammation resolution, endotoxemia, cardiac dysfunction

## Abstract

**Objective:**

Endotoxemic cardiac dysfunction contributes to greater morbidity and mortality in elderly patients with sepsis. This study tested the hypothesis that Klotho insufficiency in aging heart exaggerates and prolongs myocardial inflammation to hinder cardiac function recovery following endotoxemia.

**Methods:**

Endotoxin (0.5 mg/kg, iv) was administered to young adult (3–4 months) and old (18–22 months) mice with or without subsequent treatment with recombinant interleukin-37 (IL-37, 50 μg/kg, iv) or recombinant Klotho (10 μg/kg, iv). Cardiac function was analyzed using a microcatheter 24, 48 and 96 h later. Myocardial levels of Klotho, ICAM-1, VCAM-1 and IL-6 were determined by immunoblotting and ELISA.

**Results:**

In comparison to young adult mice, old mice had worse cardiac dysfunction accompanied by greater myocardial levels of ICAM-1, VCAM-1 and IL-6 at each time point following endotoxemia and failed to fully recover cardiac function by 96 h. The exacerbated myocardial inflammation and cardiac dysfunction were associated with endotoxemia-caused further reduction of lower myocardial Klotho level in old mice. Recombinant IL-37 promoted inflammation resolution and cardiac functional recovery in old mice. Interestingly, recombinant IL-37 markedly up-regulated myocardial Klotho levels in old mice with or without endotoxemia. Similarly, recombinant Klotho suppressed myocardial inflammatory response and promoted inflammation resolution in old endotoxemic mice, leading to complete recovery of cardiac function by 96 h.

**Conclusion:**

Myocardial Klotho insufficiency in old endotoxemic mice exacerbates myocardial inflammatory response, impairs inflammation resolution and thereby hinders cardiac functional recovery. IL-37 is capable of up-regulating myocardial Klotho expression to improve cardiac functional recovery in old endotoxemic mice.

## Introduction

Sepsis management is a major challenge for the healthcare systems worldwide. In the United States, sepsis accounts for a significant portion of all hospital deaths [1]. Sepsis occurs mainly in people of advanced age, and old septic patients have greater comorbidities and worse prognoses [2]. With the prolongation of life expectancy, the impact of sepsis is becoming greater. Investigation of the mechanisms underlying the pathobiology of sepsis in the elderly will provide information for the development of therapeutic approaches.

Septic cardiomyopathy is a common complication of severe sepsis and worsens the prognosis of patients [3]. Accumulating evidence indicates that myocardial injury caused by sepsis is due to excessive inflammation [3], and several inflammatory mediators induced by bacterial products, such as endotoxin (lipopolysaccharide, LPS), are cardiodepressants [4]. Thus, LPS contributes to the mechanism underlying the pathobiology associated with sepsis [5]. Our previous studies demonstrated that a low dose of LPS induces rapid cardiac contractile depression [6]. Further, we observed that old endotoxemic mice develop more severe cardiac dysfunction than young adult mice at 6–24 h following an exposure to LPS, along with greater elevation of cytokines and chemokines in the plasma and myocardium [6, 7]. A study found that old mice had a lower survival rate and exhibited excessive multi-organ dysfunction, as well as exaggerated inflammation during sepsis induced by cecal ligation and puncture (CLP) [8]. Observations made in laboratory animals are comparable to the clinical manifestation of older patients with sepsis. Therefore, the excessive inflammatory response may be the key risk factors for multi-organ dysfunction, particularly cardiac dysfunction, in old septic subjects.

Anti-aging protein Klotho was initially identified in the kidney [9] and has been found subsequently in the parathyroid gland, skeletal muscle and large blood vessels [7, 10, 11]. We have found that the myocardium and heart valve tissue also express Klotho, and the expression of this anti-aging protein in these cardiovascular tissues is down-regulated with aging [7, 12]. Several studies indicated that Klotho has an anti-inflammatory function [7, 13, 14]. In this regard, Klotho knockout mice display a pre-mature aging phenotype and have greater inflammation and more severe organ injury than wild-type mice when being subjected to infection [15]. More importantly, a clinical study found that renal Klotho levels were markedly reduced in septic patients [16], indicating a weakened anti-inflammatory capacity in patients with sepsis. Our previous study found that recombinant Klotho suppresses myocardial inflammatory response in the early phase of endotoxemia, up to 24 h [7]. Thus, Klotho insufficiency in aging heart likely exacerbates myocardial inflammation caused by endotoxemia and sepsis. Such pro-inflammatory effect of aging-related Klotho insufficiency may lead to worse and long-lasting cardiac dysfunction following endotoxemia or sepsis. Pharmacological up-regulation of Klotho levels in aging heart could be a feasible approach for cardiac protection in old subjects with endotoxemia or sepsis.

IL-37, a member of the IL-1 family of cytokines, was identified as an inhibitor of innate immunity [17]. Its expression has been identified in most human cell types, including monocytes, dendritic cells, endothelial cells and epithelial cells, and it acts as a natural regulator of the inflammatory responses [17]. IL-37 has repressive effects on LPS-stimulated cells, such as macrophages and endothelial cells [17]. A clinical study found that the level of IL-37 is increased in response to inflammation in old patients with sepsis [18]. In addition, our previous study found that IL-37 improves cardiac function in old mice at 24 h after an exposure to LPS [19]. Currently, the effect of IL-37 on cardiac functional recovery in the late phase of endotoxemia, at 48 to 96 h, is unclear, and the interaction of IL-37 with Klotho has not been investigated.

The purposes of this study were to determine the mechanistic role of Klotho insufficiency in myocardial inflammation and its resolution following endotoxemia in old mice and to explore the potential of IL-37 for up-regulation of myocardial Klotho level. Specifically, this study determined whether Klotho insufficiency in aging hearts leads to prolonged myocardial inflammation in endotoxemia and whether prolonged myocardial inflammation is responsible for long-lasting cardiac dysfunction. Further, this study examined whether IL-37 is capable of up-regulating Klotho level to promote myocardial inflammation resolution and cardiac function recovery in old endotoxemic mice.

## Materials and methods

### Ethics statement

The experiments were approved by the Institutional Animal Care and Use Committee of the University of Colorado Denver, and this investigation conforms to the Guide for the Care and Use of Laboratory Animals (National Research Council, revised 1996).

### Chemicals and reagents

Antibodies against ICAM-1, VCAM-1 and GAPDH were purchased from Santa Cruz Biotechnology, Inc (Dallas, TX). Antibody against Klotho was purchased from Abcam Inc. (Cambridge, MA). Recombinant Klotho protein, recombinant IL-37 protein, and Enzyme-linked immunosorbent assay (ELISA) kits for IL-6 were purchased from the R&D System (Minneapolis, MN). ELISA kit for Klotho was purchased from MyBioSource (San Diego, CA). Lipopolysaccharide (LPS, Escherichia coli 0111:B4) and all other chemicals and reagents were purchased from Sigma-Aldrich Chemical (St Louis, MO).

### Animals and treatment

Male young adult (3 to 4 months) and old (18 to 22 months) C57BL/6 mice were obtained from the Jackson Laboratory (Bar Harbor, Maine) and National Institute on Aging (Bethesda, MD), respectively. Mice were acclimated for 14 days in a 12:12 h light-dark cycle with free access to water and a regular chow diet.

Young adult mice were assigned to LPS group or saline group. Old mice were assigned to saline, LPS + saline, LPS + IL-37 or LPS + Klotho group. All treatments were performed in the morning through tail vein injection. LPS (0.5 mg/kg) was delivered in 0.1 ml of sterile normal saline. Recombinant Klotho (10 μg/kg) and recombinant IL-37 (50 μg/kg), in 0.1 ml of sterile normal saline, was administered 30–60 min after administration of LPS. Control treatment was performed using the same volume of sterile normal saline. Experiments were terminated at 24, 48 and 96 h (n ≥ 6 for each time point) after administration of LPS or saline. Blood, myocardium and kidney were collected following analysis of left ventricle (LV) function using a pressure-volume micro-catheter.

### Measurement of cardiac function

Cardiac function was assessed as described previously [20]. Briefly, mice were anesthetized with isoflurane inhalation (5% for induction, 2% for maintenance) and anticoagulated with heparin (Elkins-Sinn, Cherry Hill, NJ; 1,000 units/kg, IP). Animals were laid supine on a heating blanket and core body temperature was maintained at 37 ± 0.5°C. A pressure-volume microcatheter (Millar Instrument, Houston, TX; 1.0 F) was inserted into the left ventricle (LV) through the right common carotid artery. The pressure-volume loop and heart rate were recorded using the MPVS-400 system with the aid of PVAN software (Millar Instruments, Houston, TX). Heart rate, LV developed pressure, end-systolic and end-diastolic volume, ejection fraction, and cardiac output was analyzed.

### Immunoblotting

Immunoblotting was applied to determine Klotho protein levels in the kidney and myocardium, as well as ICAM-1 and VCAM-1 levels in the myocardium. Proteins in tissue homogenate were separated on gradient (4%−20%) mini-gels and transferred onto nitrocellulose membrane (Bio-Rad Laboratories, Hercules, CA). The membranes were blocked with 5% skim milk solution for 1 hour at room temperature. The blocked membranes were incubated with a primary antibody against a protein of interest overnight at 4°C. After washing with phosphate-buffered saline (PBS) containing 0.05% Tween 20, the membranes were incubated with a peroxidase-linked secondary antibody (specific to the primary antibody) for 1 hour at room temperature. After further washing, the membranes were treated with enhanced chemiluminescence reagents. The membrane was then exposed to X-ray films. Image J (Wayne Rasband, National Institutes of Health, Bethesda, MD) was used to analyze band density.

### ELISA

ELISA kits were utilized to quantify IL-6 and Klotho in myocardial tissue homogenate. Samples and standards were prepared according to manufacturers’ instructions. The absorbance of standards and samples was determined spectrophotometrically at 450 nm, using a microplate reader (Bio-Rad Laboratories, Inc, Hercules, CA). Results were plotted against the linear portion of a standard curve.

### Statistical analysis

Data are present as mean ± standard error (SE). Statistical analysis was performed using StatView software (Abacus Concepts, Calabasas, CA, USA). Analysis of variance (ANOVA) with Fisher post hoc test was used to analyze differences between experimental groups, and differences were confirmed using the Mann-Whitney U-test. Statistical significance was defined as P ≤ 0.05.

## Result

### Persistent cardiac dysfunction in old endotoxemic mice is associated with exacerbated and prolonged myocardial inflammation (inflamm-aging)

**Figure 1:**
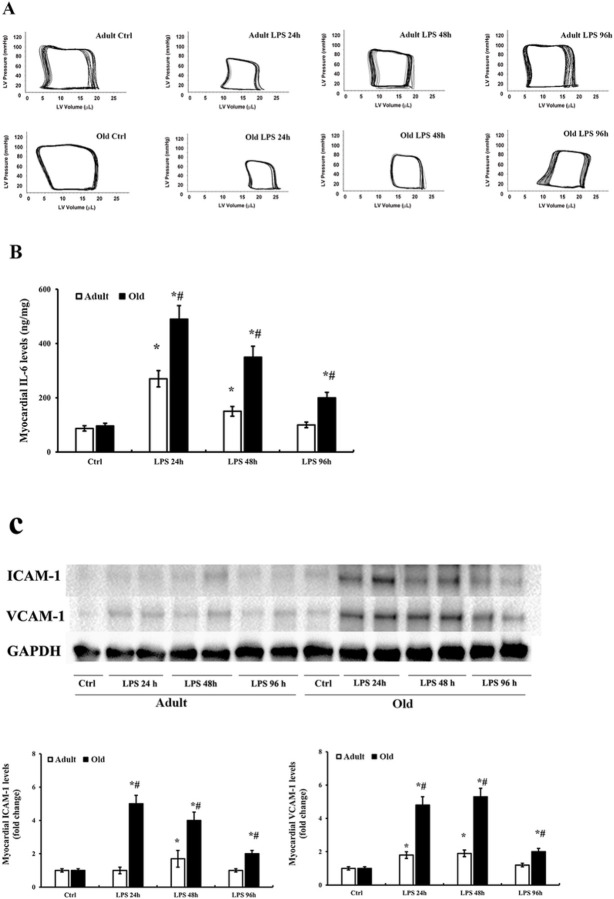
Endotoxemia induces exaggerated and prolonged inflammatory responses in hearts of old mice and impairs the recovery of cardiac function. Young adult and old mice were treated with lipopolysaccharide (LPS, 0.5 mg/kg, iv) or normal saline for 24, 48 or 96 h. Left ventricle (LV) function was analyzed using microcatheter. **(A)** Representative pressure-volume loops show that endotoxemia induced greater cardiac dysfunction at 24, 48 and 96 h in old mice compared with young adult mice. Cardiac function recovered over time in both young adult and old mice, and it was comparable to control value at 96 h in young adult mice but remained depressed in old mice at this time. Myocardial levels of IL-6 **(B)** and ICAM-1 and VCAM-1 **(C)** were assessed using ELISA and immunoblotting, respectively. Levels of these inflammatory mediators increased in the myocardium of young adult mice at 24 and 48 h, and they declined to the control level at 96 h. In old mice, myocardial levels of these inflammatory mediators were much higher at each time point than those in young adult mice, and at 96 h, they remained above control levels. Data are expressed as mean ± SE, n≥6 , *P< 0.05 vs. control, ^#^*P*< 0.05 vs. young adult at the same time point.

In previous studies on the acute effect of endotoxemia, we observed that endotoxemia induces greater myocardial inflammatory responses and worse cardiac dysfunction at 24 h in old mice compared to young adult mice [7]. To understand the relationship of cardiac functional recovery with myocardial inflammation resolution, we prolonged the observation time to 96 h in the present study. We found that both young adult and old mice displayed cardiac dysfunction at 24 h after LPS challenge. As shown in Fig. 1A and Table 1, old mice had much lower ejection fraction (EF: 31 ± 4% *vs*. 49 ± 5%; *p* < 0.05) and LV developed pressure (DP: 50 ± 5 mmHg *vs*. 62 ± 7 mmHg; *p* < 0.05) at 24 h in comparison to young adult mice. EF and DP had recovered to baseline levels at 96 h after LPS challenge in young adult mice. However, these cardiac functional parameters remained significantly lower than baseline levels in old mice (EF: 43 ± 3% *vs*. 73 ± 8%; DP: 70 ± 7 mmHg *vs*. 85 ± 9 mmHg; *p* < 0.05). These results show that endotoxin induces worse cardiac dysfunction in old mice and that the recovery of cardiac function is incomplete by 96 h in old mice.

Interestingly, we found the more severe and persistent cardiac dysfunction in old endotoxemic mice is accompanied by myocardial “inflamm-aging”, a term specifying exacerbated inflammation associated with aging [21]. As shown in Fig. 1B, myocardial IL-6 levels increased in endotoxemic young adult and old mice at 24 h. Compared with young adult mice at 24 h, myocardial IL-6 levels in old mice were about 2 folds of that in young adult mice (505 ± 67 *vs*. 260 ± 30 ng/mg; *p* < 0.05). Myocardial ICAM-1 and VCAM-1 levels were also much higher in old mice in comparison to young adult mice (Fig. 1C). The levels of IL-6, ICAM-1 and VCAM-1 in both young adult and old mice decline at 48 and 96 h. While the levels of these pro-inflammatory mediators returned to the baseline levels at 96 h in the hearts of young adult mice, they remained elevated in hearts of old mice (Fig. 1B and 1C). These results demonstrate that exaggerated myocardial inflammatory response and impaired ability of resolution of myocardial inflammation are components of myocardial inflamm-aging, and both contribute to the mechanism of persistent cardiac dysfunction in old endotoxemic mice.

### IL-37 suppresses inflammation and promotes inflammation resolution to improve the recovery of cardiac dysfunction in old endotoxemic mice

IL-37 is a member of the interleukin family and plays an anti-inflammatory effect in several inflammatory diseases. A recent study found that IL-37 improves cardiac function in adult endotoxemia mice via inhibition of the inflammatory NF-κB signaling pathway at 6 h [19]. In the present study, we found that IL-37 administration markedly improved left ventricle function in old endotoxemic mice (Table 2). Compared with the LPS only group, the developed pressure was elevated by 38%, ejection fraction was increased by 55% and cardiac output was increased by about 63% in the LPS + IL-37 group at 24 h in old mice (Table 2). Although the cardiac function was slowly recovering in both LPS alone group and LPS + IL-37 group at 48 and 96 h, the improvement was markedly improved in LPS + IL-37 group in comparison with the LPS alone group at the same time point. The left ventricle function parameters were at the baseline levels at 96 h in the LPS + IL-37 group, but not in the LPS alone group at this time point.

**Figure 2:**
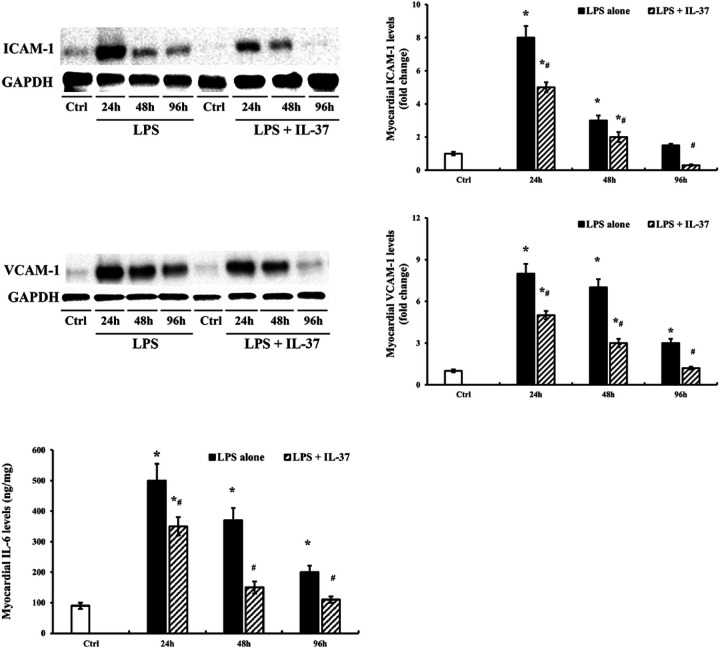
Recombinant IL-37 suppresses myocardial inflammatory responses and promotes myocardial inflammation resolution in old endotoxemic mice. Old mice were treated with LPS (0.5 mg/kg, iv) alone or LPS followed by recombinant IL-37 (50 μg/kg, iv) 30 min later. Control mice were treated with normal saline. ICAM-1 and VCAM-1 levels in myocardial tissue at 24, 48, and 96 h post-treatment were determined by immunoblotting and densitometry, and IL-6 levels were assessed by ELISA. LPS induced a significant increase in the expression of ICAM-1, VCAM-1 and IL-6 in the myocardium of old mice. Treatment with IL-37 after LPS exposure significantly reduced myocardial expression of inflammatory mediators at each time point in old mice, leading to their levels being normalized at 96 h. Data are expressed as mean ± SE, n≥6 , *P< 0.05 vs. control, ^#^*P*< 0.05 vs. LPS alone at the same time point.

Myocardial levels of IL-6, ICAM-1 and VCAM-1 were determined in the old endotoxemic mice with or without IL-37 treatment. Both ICAM-1 and VCAM-1 levels in old endotoxemic mice were reduced by 50% at 24 and 48 h by IL-37 treatment in comparison with the LPS only group. These two adhesion molecule levels returned to baseline at 96 h in the LPS + IL-37 group, but not in the LPS treatment only group. The IL-6 levels in old endotoxemic mice were decreased at 24 h and 48 h by IL-37-treatment and recovered to the baseline at 96 h (Fig. 2).

### IL-37 up-regulates myocardial expression of Klotho in old mice and preserves myocardial Klotho level following endotoxemia

**Figure 3:**
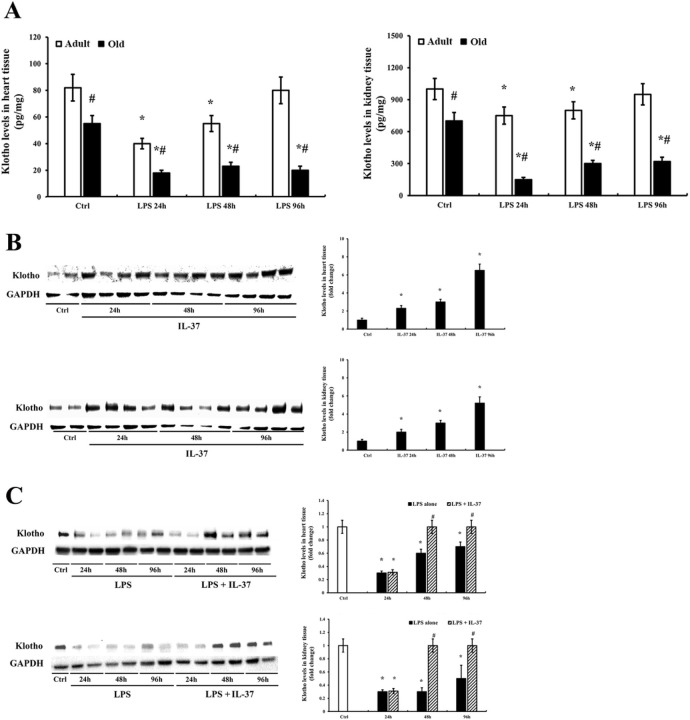
Endotoxemia causes a greater decrease in Klotho levels in the heart and kidney of old mice, and recombinant IL-37 up-regulates Klotho expression in naive old mice and promotes the restoration of Klotho levels in old endotoxemic mice. To evaluate the impact of endotoxemia on Klotho levels, young adult and old mice were treated with LPS (0.5 mg/kg, iv) for 24, 48, and 96 h. Control mice were treated with normal saline. Klotho levels in the tissue homogenate of heart and kidney were assessed using ELISA. Endotoxemia caused a significant decrease in Klotho levels in the heart and kidney. The decrease was greater in old mice **(A)**. Immunoblots and densitometry data **(B)** show that treatment of old naïve mice with recombinant IL-37 up-regulated the level of Klotho in heart and kidney in a time-dependent fashion. In addition, treatment with recombinant IL-37 30 min following LPS challenge resulted in the restoration of Klotho levels in the heart and kidney at 48 and 96 h **(C).** Data are expressed as mean ± SE, n≥6 , *P< 0.05 vs. control, ^#^*P*< 0.05 vs. young adult (in A) or vs. LPS alone (in C) at the same time point.

Figure 3A shows that Klotho levels were much lower in the heart and kidney of old mice compared with adult mice. With an exposure to endotoxin, Klotho levels markedly decreased at 24 h in the heart and kidney of both adult and old mice, and the reduction of Klotho level was greater in old mice. Interestingly, Klotho levels in the heart and kidney of young adult mice recovered to the baseline at 96 while they remained low in the heart and kidney of old mice at this time point.

To determine the effect of recombinant IL-37 on the expression of Klotho in old mice, we treated old mice with recombinant IL-37 for 24, 48 and 96 h in the absence of exposure to LPS. Surprisingly, Klotho levels in the heart and kidney were markedly elevated over time. Klotho levels in both heart and kidney at 96 h were about 5 times higher than the levels in the saline-treated control group (Fig. 3B). As LPS decreases Klotho levels in the heart and kidney of old mice at 24, 48, and 96 h, we administered recombinant IL-37 to the old endotoxemic mice. Interestingly, treatment with IL-37 prevented the decline of Klotho levels in the heart and kidney, resulting in the preservation and elevation of Klotho levels in old mice following endotoxemia (Fig. 3C).

### Klotho promotes myocardial inflammation resolution and improves the recovery from cardiac dysfunction in old mice

**Figure 4:**
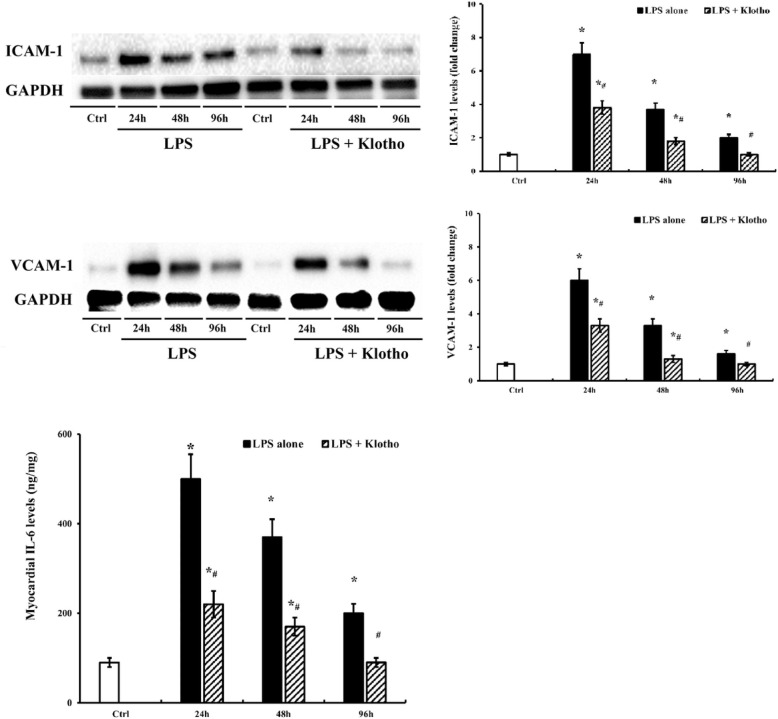
Recombinant Klotho down-regulated myocardial inflammatory responses and promotes myocardial inflammation resolution. Old mice were treated with LPS (0.5 mg/kg, iv) alone or LPS followed by recombinant Klotho (10 μg/kg, iv) 30 min later. Control mice were treated with normal saline. ICAM-1 and VCAM-1 levels in myocardial tissue at 24, 48, and 96 h post-treatment were determined by immunoblotting and densitometry, and IL-6 levels were assessed by ELISA. Treatment with recombinant Klotho reduced myocardial levels of these inflammatory mediators in old mice at 24 and 48 h following the exposure to LPS and restored their levels to the baseline by 96 h. Data are expressed as mean ± SE, n≥6 , *P< 0.05 vs. control, ^#^*P*< 0.05 vs. LPS alone at the same time point.

To determine the effect of Klotho on the myocardial inflammation resolution in old mice, we evaluated and compared the levels of ICAM-1, VCAM-1 and IL-6 levels in LPS alone group and LPS + Klotho group at 24, 48 and 96 h after administration of LPS. Although the levels of these inflammatory mediators decreased over time from 24 to 96 h, they remained significantly higher than the baseline at 96 h in LPS only group. In contrast, Klotho treatment resulted in the returning to the baseline levels for myocardial ICAM-1, VCAM-1 and IL-6 at 96 h (Fig. 4). Thus, recombinant Klotho not only attenuates myocardial inflammation response in old endotoxemic mice, but also promotes their myocardial inflammation resolution.

**Figure 5:**
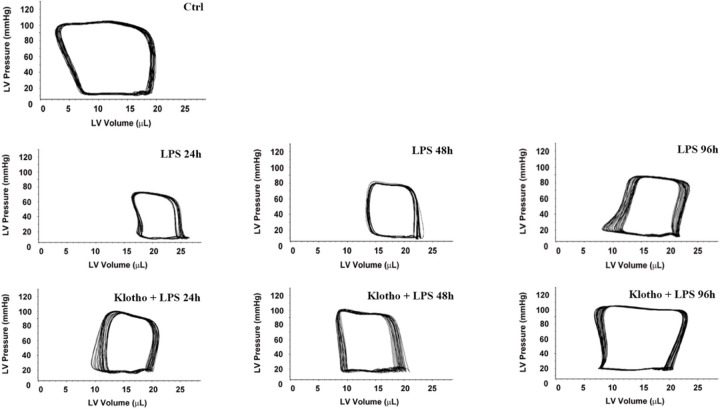
Recombinant Klotho attenuates cardiac dysfunction in old mice at 24, 48, and 96 h following exposure to LPS. Recombinant Klotho (10 μg/kg, iv) was administrated 30 min after injection of LPS (0.5 mg/kg, iv) in old mice. Control mice were treated with normal saline. Left ventricle pressure-volume loops were analyzed at 24, 48 and 96 h following LPS exposure. Klotho markedly attenuated cardiac dysfunction caused by endotoxemia in old mice at all time points. Data are expressed as mean ± SE, n≥6 , *P< 0.05 vs. control, ^#^*P*< 0.05 vs. LPS alone at the same time point.

The representative pressure-volume loops in Fig. 5 show LV function at 24, 48, and 96 h after the exposure to LPS. Compared to the saline-treated old mice, old mice treated with LPS alone displayed greater reduction in LV function at 24 h. Although LV function was improved over time, it remained significantly lower at 96 h than the control level. However, old endotoxemic mice treated with Klotho had better LV function at all time points (Fig. 5 and Table 3). At 96 h, these mice displayed significantly improved developed pressure (75 ± 6 mmHg *vs*. 68 ± 7 mmHg in LPS alone group; *P* < 0.05, n ≥ 6 ), ejection fraction (56 ± 7% *vs*. 43 ± 3% in LPS alone group; *P* < 0.05, n ≥ 6 ) and cardiac output (5.1 ± 0.6 ml/min *vs*. 4.0 ± 0.5 ml/min in LPS alone group; *P* < 0.05, n ≥ 6 ) in comparison to old mice treated with LPS alone (Fig. 5 and Table 3).

## Discussion

Organ dysfunction is common in the elderly with sepsis or endotoxemia [22]. However, it remains unclear why advanced age makes the subject more vulnerable to severe organ dysfunction. In the present study, we found that the more severe and long-lasting cardiac dysfunction in old endotoxemic mice is due to excessive and prolonged myocardial inflammation. Klotho insufficiency in the aging hearts contributes to the mechanism of exaggerated and prolonged inflammation, as well as impaired recovery of cardiac function in old endotoxemic mice. Recombinant Klotho improves the recovery of cardiac function in old endotoxemic mice by down-regulation of the myocardial inflammatory responses and promotion of inflammation resolution. Up-regulation of Klotho production in the aging heart is a novel function of anti-inflammatory cytokine IL-37 and constitutes an important mechanism by which IL-37 promotes myocardial inflammation resolution and cardiac function recovery.

The magnitude and duration of tissue inflammatory change in response to a noxious insult is determined by the level of initial inflammatory response and the ability to resolve the inflammation [23]. When the integrity of anti-inflammatory mechanism is affected by aging, inflammatory response is exaggerated and the ability of resolution of inflammation becomes attenuated, resulting in exacerbated and long-lasting inflammation. Such a phenomenon is termed as “inflamm-aging” [24]. In the present study, we observed myocardial inflamm-aging is present in old endotoxemic mice, reflected as greater magnitude of myocardial inflammation in the early phase of endotoxemia (up to 24 h after exposure to LPS) and the inability to resolve myocardial inflammation in the subsequent phase (from 24 to 96 h). Clearly, the results of the present study demonstrate that inflamm-aging exacerbates myocardial inflammation following endotoxemia by elevating the inflammatory response and attenuating the ability of clearing the inflammation.

Inflammatory cytokines, such as TNF-α, IL-1β and IL-6, are cardiodepressants and contribute to cardiac dysfunction via their direct effects on the myocardium and/or though activation of other detrimental mechanisms in the myocardium, including generation of toxic oxygen species, mitochondrial dysfunction, the loss of calcium homeostasis and expression of adhesion molecules [6, 7, 25, 26]. In the present study, we observed that in comparison to young adult mice, old mice display greater myocardial production of inflammatory mediators, i.e. IL-6, ICAM-1 and VCAM-1 examined, following an exposure to LPS, and inflammation resolution is impaired in the hearts of old mice as levels of inflammatory mediators in the myocardium remain elevated 4 days after the exposure to LPS. The exacerbated and prolonged myocardial inflammation in old endotoxemic mice is a characteristic manifestation of myocardial inflamm-aging, and it is closely associated with the more severe and long-lasting cardiac dysfunction in old endotoxemic mice. Ample evidence demonstrates the mechanistic role of inflammatory mediators in cardiac dysfunction and support the notion that inflamm-aging contributes to the mechanism underlying the more severe and persistent cardiac dysfunction caused by noxious insults in the old subjects. Currently, the mechanism of myocardial inflamm-aging is unclear.

Klotho is an anti-aging protein present in multiple tissues, and its level declines with aging. Decreased Klotho protein level correlates with reduced lifespan and aging-related disorders, such as osteoporosis, coronary artery disease, and stroke [27]. Indeed, we observed significantly lower levels of myocardial Klotho in old mice in comparison to young adult mice. Interesting observations of the present study include greater reduction of myocardial Klotho levels by endotoxemia in old mice and inability of aging hearts to restore their Klotho levels 4 days after in endotoxemia. A low level of inflammation associated with aging is one of the mechanisms of Klotho down-regulation [28]. The greater reduction of myocardial Klotho level and failure to restore the Klotho level after 4 days are likely due to exaggerated production of inflammatory mediators and persistent elevation of these mediators in the myocardium as inflammatory cytokines have been found to down-regulate Klotho expression in mice [29]. Several studies demonstrate that Klotho has an anti-inflammatory function[7, 13, 30]. Particularly, recombinant Klotho has been found to inhibit TNF-α-induced NF-κB activation and attenuate the expression of ICAM-1 and VCAM-1 in HUVECs [31]. Based on the information, it is reasonable to hypothesize that Klotho insufficiency in aging hearts and further reduction myocardial Klotho level following endotoxemia play a mechanistic role in mediating myocardial hyper-inflammatory response and persistent myocardial inflammation observed in old endotoxemic mice.

We tested this hypothesis by evaluating the effect of recombinant Klotho on myocardial inflammation and cardiac function in old endotoxemic mice. Recombinant Klotho not only reduced the levels of inflammatory mediators in the myocardium at 24 h, but also promoted the resolution of myocardial inflammation, resulting in essentially normalized levels of inflammatory mediators in the myocardium after 4 days into endotoxemia. As a result, cardiac function recovery is markedly improved to a level slightly lower than control. The novel finding that Klotho promotes myocardial inflammation resolution and cardiac function recovery in old endotoxemic mice indicates that Klotho insufficiency is responsible for the impaired myocardial inflammation resolution and delayed cardiac function recovery. This finding also highlights the therapeutic potential of recombinant Klotho for cardiac protection in old subjects affected by endotoxemia or sepsis.

Inflammation associated with aging is believed to be a mechanism by which Klotho expression is down-regulated in old subjects [28]. However, it remains unclear whether Klotho expression in old subjects could be up-regulated. We explored the potential of anti-inflammatory approach for up-regulation of Klotho expression in old mice.

Our previous study demonstrates a potent anti-inflammatory effect of IL-37 on the cardiovascular tissue [19, 32]. Interestingly, we observed in the present study that administration of recombinant IL-37 to naïve old mice increases Klotho protein levels in the heart and kidney in a time-dependent fashion, with the maximal increase at 4 days. Further, treatment of old endotoxemic mice with recombinant IL-37 results in the restoration of Klotho levels in the heart and kidney at 4 days into endotoxemia. With the restoration of Klotho level in the heart, myocardial inflammation is completely resolved, and cardiac function is fully recovered in IL-37-treated old endotoxemic mice at 4 days into endotoxemia. It is noteworthy that old endotoxemic mice treated with recombinant IL-37 have better cardiac function than those treated with recombinant Klotho. It is likely that other mechanisms utilized by IL-37 in addition to the mechanism through up-regulation of Klotho have a moderate contribution to the full recovery of cardiac function.

Anti-inflammation might be the main mechanism by which IL-37 up-regulates Klotho levels. The effect IL-37 on Klotho level appears to be systemic, not selective to the heart, as kidney Klotho levels are also increased in old mice treated with IL-37. Up-regulation of Klotho by IL-37 occurs in both naïve old mice and old endotoxemic mice, demonstrating a novel mechanism for protection of the heart against aging-related inflammatory conditions. The beneficial effect of recombinant IL-37 on Klotho expression in naïve old mice is especially significant as it indicates that this approach may have a broader application for anti-aging and prevention of aging-related health problems.

Noticeably, myocardial inflammation is completely resolved, and cardiac function is fully recovered in young adult mice 4 days after endotoxemia while myocardial inflammation is still present and cardiac function remains low in old mice at this time. However, cardiac dysfunction in old endotoxemic mice appears to be reversible as the recovery is markedly improved by recombinant Klotho or IL-37. It is unclear from the present study how many more days are needed for the complete inflammation resolution and full cardiac function recovery in old endotoxemic mice without intervention. Our preliminary experiment indicates that approximately 10 days are required. Future study will determine the time course of natural recovery from endotoxemia in old mice and the days shortened by therapeutic interventions.

## Conclusion

**Figure 6:**
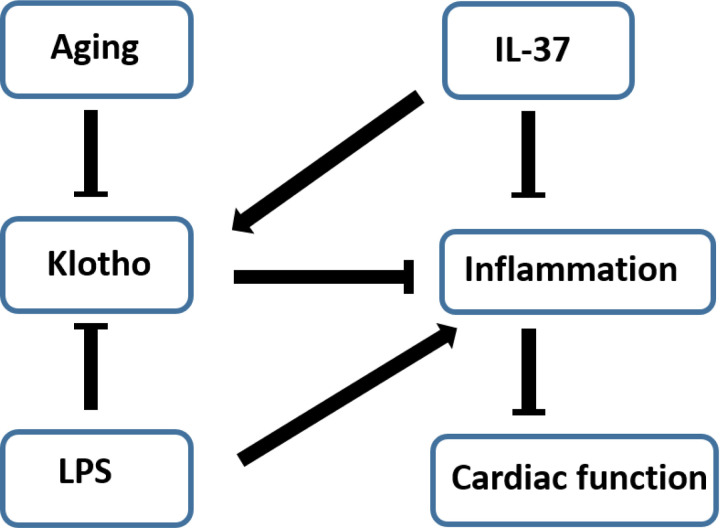
Schematic diagram summarizes the main findings of this study. While aging down-regulates Klotho expression to augment myocardial inflammation and cardiac dysfunction in endotoxemia, IL-37 up-regulates Klotho expression to suppress myocardial inflammatory responses and to promote myocardial inflammation resolution. The direct effect of IL-37 on the inflammatory responses and indirect effect through induction of Klotho promote inflammation resolution and cardiac function recovery in old endotoxemic mice.

The present study demonstrates that exacerbated the persistent inflammation in old endotoxemic mice contributes to the exaggerated and prolonged cardiac dysfunction. Klotho insufficiency in the aging heart plays a mechanistic role in mediating myocardial hyper-inflammation and more severe cardiac dysfunction in old endotoxemic mice. Recombinant Klotho suppresses myocardial inflammatory response, promotes myocardial inflammation resolution and improves cardiac function recovery. Recombinant IL-37 is capable of up-regulating Klotho expression in the heart and kidney of old naïve mice, abrogating the delay in inflammation resolution and cardiac function recovery in old endotoxemic mice (Fig. 6). Both Klotho and IL-37 have the potential for cardiac protection in old subjects affected by endotoxemia or sepsis.

## Figures and Tables

**Table 1:** Old mice display more severe cardiac dysfunction following endotoxemia

	Adult Ctrl	Adult LPS 24 h	Adult LPS 48 h	Adult LPS 96 h	Old Ctrl	Old LPS 24 h	Old LPS 48 h	Old LPS 96 h
Heart rate (bpm)	450 ± 38	560 ± 45*	520 ± 54	490 ± 41	445 ± 51	540 ± 52*	515 ± 51	470 ± 60
Developed Pressure (mmHg)	85 ±6	53 ± 6*	72 ± 5*	83 ± 9	83 ± 8	43 ± 5*^#^	60 ± 5*^#^	65 ± 7*^#^
End-systolic volume (ul)	5.8 ± 0.6	11.5 ± 0.9*	8.5 ± 1.1*	5.8 ± 0.7	6.9 ± 0.7	18.0 ± 1.5*^#^	15.8 ± 1.2*^#^	12.1 ± 1.1^#^
End-diastolic volume (ul)	18.0 ± 2.0	20.0 ± 2.3	18.0 ± 1.9	17.5 ± 1.3	18.0 ± 1.3	25.0 ± 2.0*^#^	24.1 ± 2.3^#^	22.2 ± 1.7*^#^
Ejection Fraction (%)	73 ± 7	47 ± 5*	55 ± 6*	65 ± 7	68 ± 7	30 ± 4*^#^	37 ± 4*^#^	43 ± 5*^#^
Cardiac output (ml/min)	6.3 ± 0.5	4.8 ± 0.5*	5.1 ± 0.5*	5.8 ± 0.4	6.0 ± 0.4	3.3 ± 0.3*^#^	3.8 ± 0.3*^#^	4.4 ± 0.4*^#^

Data are expressed as mean ± SE. n ≥ 6 in each group;

* *P* < 0.05 vs. control,

# *P* < 0.05 vs. young adult at the same time point.

**Table 2:** Recombinant IL-37 improves cardiac functional recovery in old mice following endotoxemia

	Ctrl	LPS 24 h	LPS 48 h	LPS 96 h	LPS + IL-37 24 h	LPS + IL-37 48 h	LPS + IL-37 96 h
Heart rate (bpm)	480 ± 45	540 ± 60	510 ± 65	490 ± 36	510 ± 62	480 ± 42	485 ± 57
Developed Pressure (mmHg)	80 ± 7	50 ± 5*	58 ± 7*	68 ± 7*	69 ± 7*^#^	71 ± 8*^#^	76 ± 9^#^
End-systolic volume (ul)	7.0 ± 0.6	18.5 ± 0.7*	14.5 ± 1.3*	11.0 ± 1.2*	10.0 ± 1.2*^#^	8.9 ± 0.9*^#^	7.1 ± 0.5^#^
End-diastolic volume (ul)	18.0 ± 2.0	25.0 ± 2.6*	22.0 ± 2.1*	20.0 ± 1.9*	20.0 ± 1.8*^#^	19.0 ± 2.0^#^	18.2 ± 1.8^#^
Ejection Fraction (%)	65 ± 7	31 ± 4*	35 ± 3*	43 ± 3*	48 ± 6*^#^	50 ± 6*^#^	60 ± 7^#^
Cardiac output (ml/min)	5.5 ± 0.6	3.0 ± 0.4*	3.3 ± 0.4*	4.0 ± 0.5*	4.9 ± 0.5*^#^	5.0 ± 0.6*^#^	5.3 ± 0.5^#^

Data are expressed as mean ± SE. n ≥ 8 in Ctrl and LPS alone groups; n ≥ 6 in LPS + IL-37 groups;

* *P* < 0.05 vs. control,

# *P* < 0.05 vs. LPS alone at the same time point.

**Table 3:** Recombinant Klotho improves cardiac functional recovery in old mice following endotoxemia

	Ctrl	LPS 24h	LPS 48h	LPS 96h	LPS + Klotho 24 h	LPS + Klotho 48 h	LPS + Klotho 96 h
Heart rate (bpm)	480 ± 45	540 ± 60	510 ± 65	490 ± 36	520 ± 49	490 ± 53	480 ± 38
Developed pressure (mmHg)	80 ± 7	50 ± 5*	58 ± 7*	68 ± 7*	65 ± 8*^#^	70 ± 9*^#^	75 ± 6
End-systolic volume (ul)	7.0 ± 0.6	18.5 ± 0.7*	14.5 ± 1.3*	11.0 ± 1.2*	12.7 ± 1.3*^#^	9.0 ± 1.0*^#^	8.0 ± 0.7^#^
End-diastolic volume (ul)	18.0 ± 2.0	25.0 ± 2.6*	22.0 ± 2.1*	20.0 ± 1.9*	21.1 ± 1.8*^#^	19.0 ± 2.1	18.0 ± 2.0
Ejection Fraction (%)	65 ± 7	31 ± 4*	35 ± 3*	43 ± 3*	44 ± 5*^#^	50 ± 6*^#^	56 ± 7^#^
Cardiac output (ml/min)	5.5 ± 0.6	3.0 ± 0.4*	3.3 ± 0.4*	4.0 ± 0.5*	4.0 ±0.3*^#^	5.0 ± 0.5*^#^	5.1 ± 0.6^#^

Data are expressed as mean ± SE. n ≥ 8 in Ctrl and LPS alone groups; n ≥ 6 in LPS + Klotho groups;

* *P* < 0.05 vs. control,

# *P* < 0.05 vs. LPS alone at the same time point.
